# 4375 Developing team science for practical applications of artificial intelligence in health systems to improve value and outcomes: A case study in reducing avoidable emergency department use

**DOI:** 10.1017/cts.2020.355

**Published:** 2020-07-29

**Authors:** Vladimir G. Manuel, Eran Halperin, Jeffrey Chiang, Kodi Taraszka, Laura Kim, Naveen Raja, Christopher Saigal, Lily Roh, Eleazer Eskin

**Affiliations:** 1 David Geffen School of Medicine at UCLA; 2 University of California, Los Angeles

## Abstract

OBJECTIVES/GOALS: Health care systems are complex, dynamic, and varied. Advances in artificial intelligence (AI) are enabling healthcare systems to use their own data to elicit patterns and design suitable interventions. To realize this potential, computer scientists and clinicians need an effective, practical, and replicable approach to collaboration METHODS/STUDY POPULATION: In this study, computer scientists partnered with clinicians to investigate predictors of avoidable emergency department use. The team sought an approach to computational medicine that could increase the relevance and impact of prediction to solve pressing problems in the health system. The team adopted an emergent architecture that engaged system leaders, computer scientists, data scientists, health services researchers, and practicing clinicians with deep ambulatory and inpatient knowledge to form the initial questions that shaped the prediction model; to understand nuances of coding and recording in source data and the implications for models; and to generate insights for promising points of intervention. The team recorded decisions and challenges as it progressed to analyze its function. RESULTS/ANTICIPATED RESULTS: Most avoidance models focus on a narrow time period around target events, or on high cost patients and events. This interdisciplinary team used their insights into the health system’s workflows and patient population to adopt a longitudinal approach to their prediction models. They used AI to build models of behavior in the system and consider prevention points across clinical units, time, and place. The holistic, systemwide focus enabled the team to generate insights that the system leaders and subsequently specific clinical units could apply to improve value and outcomes. A facilitated team process using learning system and cooperative network principles allowed a large and modular interdisciplinary team to build a transparent AI modeling process that yielded actionable insights into hypercomplex workflows. DISCUSSION/SIGNIFICANCE OF IMPACT: An architecture for involving diverse stakeholders in computational medicine projects can increase the relevance and impact of AI for solving care delivery problems in complex health systems. Translational science and computational medicine programs can foster this type of engagement and encourage a whole system perspective.

